# Behavioral plasticity and G × E of reproductive tactics in *Nicrophorus vespilloides* burying beetles

**DOI:** 10.1111/evo.12619

**Published:** 2015-03-10

**Authors:** Mauricio J. Carter, Megan L. Head, Allen J. Moore, Nick J. Royle

**Affiliations:** ^1^ Centre for Ecology and Conservation, College of Life and Environmental Sciences, University of ExeterCornwall CampusPenrynTR10 9EZUnited Kingdom; ^2^ Division of Evolution, Ecology and Genetics, Research School of BiologyThe Australian National UniversityCanberraACT2601Australia; ^3^ Department of GeneticsUniversity of GeorgiaAthensGeorgia30602

**Keywords:** Alternative reproductive tactics, conditional strategy, contest behavior, male–male competition, phenotypic plasticity

## Abstract

Phenotypic plasticity is important in the evolution of traits and facilitates adaptation to rapid environmental changes. However, variation in plasticity at the individual level, and the heritable basis underlying this plasticity is rarely quantified for behavioral traits. Alternative behavioral reproductive tactics are key components of mating systems but are not often considered within a phenotypic plasticity framework (i.e., as reaction norms). Here, using lines artificially selected for repeated mating rate, we test for genetic (G × E) sources of variation in reproductive behavior of male *Nicrophorus vespilloides* burying beetles (including signaling behavior), as well as the role of individual body size, in responsiveness to changes in social environment. The results show that body size influences the response of individuals’ signaling behavior to changes in the social environment. Moreover, there was G × E underlying the responses of males to variation in the quality of social environment experienced (relative size of focal male compared to his rival). This shows that individual variation in plasticity and social sensitivity of signaling behavior can evolve in response to selection on investment in mating behavior, with males selected for high mating investment having greater social sensitivity.

Phenotypic plasticity, the environmentally sensitive production of alternative phenotypes by given genotypes (DeWitt and Scheiner 2004), is a common, but relatively poorly understood, feature of organisms (Gomez‐Mestre and Jovani 2013). Models show that fluctuating or rapid directional change in the environment is expected to provide strong selection for plasticity, which can increase the speed at which populations respond to environmental change and maintain genetic variation, so promoting the persistence of populations (Gomez‐Mestre and Jovani 2013). Phenotypic plasticity can be either irreversible (developmental plasticity) or reversible (i.e., behavioral and/or physiological plasticity), and is described using reaction norms (Piersma and Drent 2003). The majority of studies of phenotypic plasticity have focused on irreversible (developmental) plasticity, but it has more recently been recognized that reversible plasticity, especially behavioral plasticity, may be central to understanding organismal adaptation (Nussey et al. 2007; Dingemanse et al. 2010; Dingemanse and Wolf 2013; Foster 2013).

Population responses to environmental variation will depend upon plasticity at the level of the individual (Nussey et al. 2007; Han and Brooks 2014) and a behavioral response is usually the first response of organisms to a rapid change in their environment (Mayr 1963). However, despite its potential importance individual‐level plasticity of behavioral traits, and, more importantly, whether this plasticity has a heritable basis (i.e., genotype by environment interactions; G × E) is rarely measured (Nussey et al. 2007; Dingemanse and Wolf 2013; but see Taylor et al. 2013; Bretman et al. 2014 for notable exceptions). Behavioral plasticity allows individuals to adjust their behavior to rapid changes in their current environment and is expected to evolve when there is a fitness advantage over less plastic individuals (Dingemanse and Wolf 2013). Such reversible plasticity is expected when environmental variation is predominantly fine grained (varies within the lifetime of an individual) rather than coarse grained (varies primarily between, rather than within, generations) (Snell‐Rood 2013), and can evolve when there is heritable variation underlying plasticity (G × E; Nussey et al. 2007).

Alternative reproductive tactics are a key component of many mating systems (Hazel et al. 1990; Gross 1996) and provide a potential example of phenotypic plasticity. However, alternative reproductive tactics are rarely explicitly considered within the framework of phenotypic plasticity (Carroll and Corneli 1995; Bretman et al. 2011a; Neff and Svensson 2013). Instead, theoretical treatments of the evolution and maintenance of alternative reproductive strategies typically present a dichotomy between “genetic” causes (fixed or mixed strategies) and “environmental” causes (conditional strategies) of phenotypic variation in mating tactics that make up an individual's overall reproductive strategy (Dawkins 1980; Dominey 1984; Gross 1996; Tomkins and Hazel 2007). However, as is true for other phenotypic traits, both genetic and environmental variation are likely to be important in determining the expression of reproductive tactics (Neff and Svensson 2013) and alternatives may be expressed as reversible (e.g., behavioral), not just as irreversible (developmental) traits.

When G × Es influence alternative reproductive strategies, discrete alternative reproductive tactics reflect a threshold trait whose expression depends on both the social environment (E) experienced by the individual (Hazel et al. 2004; Tomkins and Hazel 2007; Neff and Svensson 2013), and genetic variation (G) for the switch point between tactics (Roff 1996). This approach has been used to investigate the expression of alternative reproductive tactics of irreversible (discontinuous) morphological traits (Tomkins and Brown 2004; Buzatto et al. 2010; Tomkins et al. 2011), but there has been considerably less attention given to quantifying genetic variation in the expression of alternative reproductive tactics of reversible, behavioral, traits (Neff and Svensson 2013; although see Carroll and Corneli 1995). To understand the maintenance and evolution of alternative behavioral tactics, and behavioral plasticity in general, it is therefore important to identify sources of individual and genetic variation in response to changes in the environment experienced by individuals on the expression of behaviors involved in these reproductive tactics (West‐Eberhard 1989).

In the current study we test for genetic (G × E) sources of variation in reproductive behavior (i.e., behavioral plasticity of reproductive tactics) in response to changes in the social environment experienced by individuals, using burying beetles (*Nicrophorus vespilloides)* as a model. Male burying beetles actively compete with each other for access to breeding resources (i.e., small vertebrate carcasses), and when they find a suitable carcass will emit pheromones as a signal to attract females. Emission of pheromones, as well as odors from the carcass, may also attract other males. As a consequence, intrasexual competition is common and usually leads to the establishment of a dominant male and female pair, or “resource holders,” who process the carcass and use it for breeding (Pukowski 1933; Eggert and Müller 1989a). However, when there is more than one individual of a given sex at a carcass, females and males who are not resource holders can adopt alternative, subordinate, reproductive tactics to increase the probability of reproductive success (Eggert and Müller 1989a, b, 1997). Subordinate females may lay eggs near the carcass (brood parasites) and subordinate males adopt a “satellite” tactic and attract females by signaling (emitting pheromones) off the carcass (Eggert and Müller 1989a). Satellite males sneak copulations with females (Eggert and Müller 1989a; Müller et al. 1998; Pettinger et al. 2011) and all females appear to be multiply mated (Pettinger et al. 2011), creating uncertainty over paternity (Sakaluk and Müller 2008).

Previous work has shown that the expression of alternative tactics is reversible (Eggert and Müller 1989a), conditional on the size of the focal male (Beeler et al. 1999; Walling et al. 2008). Larger males are generally competitively superior (dominant) to smaller males (Müller et al. 2007; Hopwood et al. 2014), and large males are more likely to display “resource‐holder” behaviors than small males (e.g., signaling on the carcass) in the absence of competitors (Beeler et al. 1999; Walling et al. 2008). There is also evidence that such behaviors have a heritable basis (Eggert 1992). However, G × E of the expression of alternative reproductive tactics in burying beetles have not been explicitly quantified, especially the change of reproductive behavior in response to the arrival of a competitor male (i.e., a change in the social environment; but see Sakaluk and Müller 2008 for an example of male behavioral plasticity in duration of copulation in response to social environmental variation). Such rapid changes in the social environment are a central feature of burying beetle natural history (Scott 1998; Sakaluk and Müller 2008; Hopwood et al. 2014).

Here we used artificial selection on repeated mating rate, a paternity assurance trait in *N. vespilloides* beetles (Head et al. 2014), to examine how variation in individual characteristics (body size) and genetic background (line) interact with social environmental effects to determine the expression of alternative reproductive tactics (i.e., “resource‐holder” vs. “satellite” behaviors). In general, male social plasticity in the context of reproduction is expected to be greater when there is increased mating competition and when female control over mating is low (Bretman et al. 2011a). Male burying beetles respond to increased mating competition by increasing their repeated mating rate (House et al. 2008; Sakaluk and Müller 2008), and control over mating rate is primarily determined by male behavior in *N. vespilloides* (Head et al. 2014). Consequently, we predict that males artificially selected for high repeated mating rate will be more sensitive to variation in the social environment that they experience than males selected for low mating rate (see also Sih et al. 2014). In addition, using artificial selection lines allows us to not only identify genetic effects underlying behavioral plasticity but also provides us with information on how selection shapes these traits (e.g., a response of signaling behavior to selection on investment in mating rate indicates that these traits are genetically correlated) (Müller et al. 1998). To examine the effects of social environment on the expression of alternative reproductive behaviors, we recorded male investment in resource holding behaviors in general (the proportion of time spent on the carcass) in addition to each behavioral tactic (the proportion of time spent signaling on the carcass vs. signaling off the carcass) in both the absence and presence of a competitor. This allowed us to determine whether signaling behavior is plastic in response to a change in the social environment per se as well as establishing the role of body size in moderating the degree of behavioral plasticity. By assessing the behavior of males from different selection regimes in different social environments, we are also able to investigate the effects of genotype‐by‐environment interactions on the expression of behaviors associated with alternative reproductive tactics. Furthermore, we also examined whether the quality of the social environment experienced by focal males (i.e., the size of males in relation to their competitor) affected the expression of behaviors, and whether there is any genetic variation underlying this response.

We predicted that

- Focal males would change their behavior when a competitor was introduced (i.e., there is behavioral plasticity).
- Relative body size would mediate this response, with larger males investing more in resource‐holding behaviors than small males.
- There would be underlying genetic variation for this behavioral plasticity (i.e., there would be a significant selection regime × social environment interaction), with males selected for high mating rates more socially sensitive to variation in the social environment than males selected for low mating rates.

## Material and Methods

### EXPERIMENTAL SETUP

Beetles used in this experiment were obtained from generation 12 of lines selected for high and low mating rate (Supporting Information; for details of selection regime, origin, and maintenance of beetles see Head et al. 2014). Focal males used in our experiment were sexually mature, unmated virgins 13–15 days post eclosion. We setup 40 males per replicate line (*N* = 160 in total), but mortality in either focal or competitor males prior to observations resulted in slightly reduced sample sizes (High 1 = 31; High 2 = 39; Low 1 = 30; Low 2 = 38; final sample size = 139). We did not use males from the control selection lines in our experiment because we were explicitly interested in how divergence in mating rate influences reproductive tactics. Prior to behavioral observations, all males were kept in individual containers (clear plastic container: 7 × 7 × 4 cm) filled with 2 cm of moist soil, in conditions identical to those experienced during selection. Competitor males were taken from a wild‐derived stock population, which had been maintained in the laboratory for two generations. These males were also housed individually before being used in the experiment. Prior to being added to experimental containers, we determined the mass (to 0.0001 g using an Ohaus Explorer balance) and size (pronotum width; to 0.1 mm using dial callipers) of both focal and competitor males. Males were then placed in an experimental container, which consisted of a transparent plastic box (17 × 11 × 6 cm) containing 1 cm of moist soil and a 15–25 g freshly thawed mouse carcass. Competitor males were given a white mark on the top of their pronotum to facilitate male identification. Marking does not affect behavior (Hopwood et al. 2013).

### EXPERIMENTAL DESIGN

To investigate the genetic basis of behavioral plasticity, we observed behavior of focal males from different genetic backgrounds (selection regimes) when by themselves and in the presence of a rival male (competitor). Two approaches were used to evaluate dominant males (resource holders) and subordinate males (satellite). First, we define the total amount of time that focal males spend on the carcass (general activity) as a proxy of “resource‐holding” behavior. General activity included signaling behavior, eating, self‐grooming, and walking around on the carcass (Beeler et al. 1999). Second, we evaluated male signaling behavior itself. In burying beetles this involves the emission of pheromones and is easily observed as males adopt a stereotypical “headstand” posture, lifting and extending their abdomens into the air to emit pheromones (Beeler et al. 1999). Dominant males (resource holders) spend more of their time signaling on the carcass, whereas subordinate males spend more time signaling off the carcass (satellites) (Eggert and Müller 1989a).

The signaling (emission of pheromones) and general activity of each male was monitored by scan sampling every 10 min for 4 h prior to the onset of the scotophase, the time of maximal signaling effort in this species (Walling et al. 2008). Male position was recorded (on‐carcass, off‐carcass, or not visible) and, if visible, what behaviors they were engaged in (signaling, or other behavior such as eating, self‐grooming, walking). We randomly divided focal males from each replicate line into four groups for observation. For each group, 40 males were placed in individual experimental boxes in an incubator with controlled temperature (20°C) and photoperiod (18 light–6 dark). Focal males were added to the container with the breeding resource (mouse carcass) and left alone for 24 h to establish residency. The behavior of these focal males was then first observed in the absence of a competitor. The following morning a competitor male was added to the experimental container and the two males were allowed to acclimate and interact for a further 24 h before focal male behavior was recorded. This protocol of staggering the arrival of males replicates male arrival behavior at carcasses in the wild (Hopwood et al. 2014).

The data collected allowed us to quantify G × E of behavioral plasticity in the context of two key questions: (1) how does focal male behavior change in response to the arrival of a competitor? (2) Is this change sensitive to variation in the quality of the social environment experienced (i.e., his size relative to that of the rival male; Hopwood et al. 2014)? To address the first question, the social environment was defined as a binary variable (rival male present/absent) with the presence of G × E for behavioral plasticity indicated by a significant interaction between selection regime (G) and social environment (E). To address the second question, we used the trials where focal males were signaling in the presence of a competitor only. G × E on the expression of signaling behavior in response to variation in the quality of the social environment experienced was determined by a significant interaction between the selection regime (G) and the size of focal male relative to that of his rival (E). Competitor males were randomly drawn from the stock population, which did not differ in mean or variation in size from the population of focal males (focal male pronotum width = 4.98 mm ± 0.04 (CI), competitor male pronotum width = 5.01 mm ± 0.04 (CI); *F*_1,606_ = 0.90, *P* = 0.34). This generated variation in the social environment experienced by focal males; from good (focal male larger than competitor) to poor (focal male smaller than competitor). Previous work has shown that relative male size is the most important determinant of contest outcomes over carcasses (Hopwood et al. 2013, 2014; Lee et al. 2014). Our experimental design replicated what is likely to happen in the wild, where there is uncertainty about the size of competitors that males will encounter in competition for breeding resources (Hopwood et al. 2014).

### STATISTICAL ANALYSIS

In the first analysis, we examined the effects of genetic background (selection regime; G) and social environment (presence or absence of competitor; E) on the behavioral plasticity of the proportion of time spent active on the carcass (“resource defense” behavior) and signaling behavior using general linear mixed models with a Gaussian error structure and square root transformed response variables (GLMMs). Selection regime (high or low mating rate) and social environment (presence/absence of a competitor) were used as explanatory variables, with the interaction between the two included in models. Replicate nested within selection regime and male focal identity (ID) were included as random effects, and body size of focal males (pronotum width, P) was also included as a covariate (fixed effect). Response variables were (1) the proportion of time spent active (signaling and/or engaged in other behaviors) on the carcass, (2) the proportion of active time spent signaling on the carcass, and (3) the proportion of active time spent signaling off the carcass.

In the second analysis, we examined the effect of the quality of the social environment experienced (i.e., size relative to competitor; E) and genetic background (selection regime; G) on expression of signaling behavior. We used the relative size difference in pronotum width between focal and competitor males as the measure of competitive ability (E) (Hopwood et al. 2014), and selection regime (high or low mating rate; G), and their interaction, as explanatory variables. Replicate nested within selection regime was included as a random effect, and binomial errors were used to fit the models. Similar to the plasticity analysis above response variables for models were (1) the proportion of time spent signaling on the carcass, and (2) the proportion of time spent signaling off the carcass.

We used sequential stepwise removal of terms to test the significance of fixed factors in the models. Comparisons between models were made using analysis of deviance (the −2log likelihood and χ^2^ with the degrees of freedom depending on the variables removed from the models). Results are presented with means and ±95% CIs obtained from the analyses. All analyses were performed using “R” version 3.0.2 (R Development Core Team 2011).

## Results

### PLASTICITY OF “RESOURCE DEFENSE” BEHAVIOR

Focal males (who were introduced to the carcass before competitor males) spent a greater proportion of their time on the carcass, following the introduction of competitor males, than their rivals (focal males: 0.85 ± 0.013 (CI), competitor males: 0.72 ± 0.021 (CI); GLM with quasibinomial errors, *n* = 309; *F*_1,307_ = 15.705, *P* < 0.0001).

**Figure 1:**
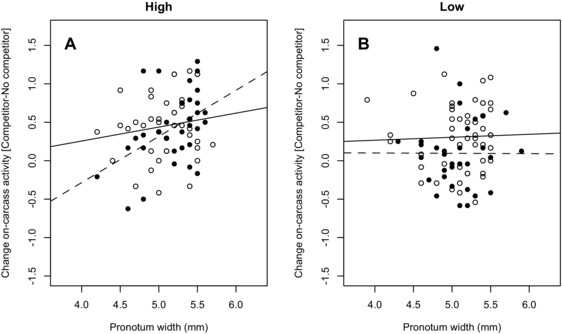
Change in the time spent active on the carcass plotted against focal male body size for each selection regime and trial. Open circles correspond to males in the “no competitor” scenario, solid circles correspond to males in the “competitor” scenario. Regression lines were obtained from the statistical analyses as indicated in the text.

**Figure 2:**
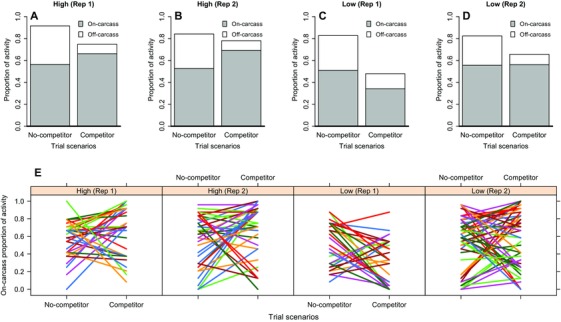
Proportion of time spent active on the carcass and off the carcass when no competitor was present and when a competitor was present in relation to selection regime (high—A, B; low—C, D), and associated individual reaction norms for the amount of time focal males spent on the carcass (E).

The proportion of time focal males spent on the carcass (signaling and engaged in other behaviors) depended upon an interaction between selection regime (G) and social environment (E) and an interaction between the size of the focal male (P) and the social environment (E) (Table 1). In the first interaction males from lines selected for high mating rates increased the amount of time spent on the carcass when a competitor was introduced more than males from low lines (i.e., there was a G × E for plasticity of time spent on the carcass; Figs. 1, 2A–D). In the latter interaction when there was no competitor present, the time spent on the carcass was independent of focal male size, but when a competitor was introduced large males increased the amount of time spent on the carcass whereas small males spent less time on the carcass (i.e., the direction of change in time spent on the carcass depended on the size of the focal male involved; Fig. 1). The interaction between selection regime and focal male size was nonsignificant (Table 1, Fig. 1) as were the main effects of social environment and selection regime (Table 1, Fig. 2A–D), but there was a significant overall, positive, main effect of focal male body size (Table 1). When the random effect ID was removed from the original model, this did not significantly alter the fit of the model ($\chi_{2}^{2}=5.21,P=0.073$), indicating considerable variation among and within males in their response to a change in social environment (Fig. 2E).

**Table 1:** Mixed model analyses testing plasticity in “resource defence” and signaling behaviors

On‐carcass activity		
	$x_{1}^{2}$	*P*‐value
Pronotum width (P)	7.444	0.006
Selection regime (G)	3.537	0.059
Social environment (E)	0.001	0.966
P × G	0.437	0.508
P × E	4.776	0.028
G × E	5.147	0.023
P × G × E	3.029	0.081
On‐carcass signaling behavior		
Pronotum width (P)	7.591	0.005
Selection regime (G)	1.936	0.164
Social environment (E)	6.547	0.011
P × G	0.123	0.725
P × E	3.442	0.063
G × E	3.254	0.071
P × G × E	3.724	0.053
Off‐carcass signaling behavior		
Pronotum width (P)	2.903	0.088
Selection regime (G)	0.197	0.657
Social environment (E)	42.61	<0.0001
P × G	0.125	0.723
P × E	0.364	0.546
G × E	0.273	0.601
P × G × E	3.724	0.053

*x* ^2^ Values to test the significance of the fixed effects were obtained by log‐likelihood ratio test following sequential stepwise removal of terms.

### PLASTICITY OF SIGNALING BEHAVIOR

**Figure 3:**
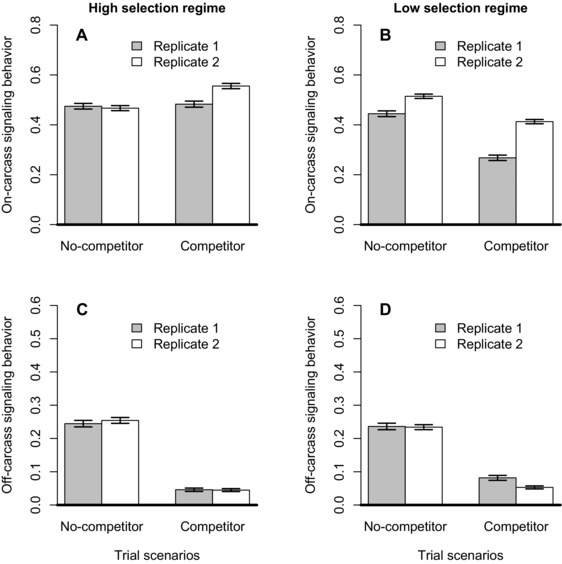
Proportion of time spent signaling on (A, B) and off (C, D) the carcass without a competitor and with a competitor. Signaling behavior of males from high mating rate selection regimes shown in A and C (mean ± 95% CIs). Signaling behavior of males from low mating rate selection regimes shown in B and D (mean ± 95% CIs).

As expected, when a competitor male was present signaling behavior both on‐ and off‐carcass changed compared to when there was no competitor present (Fig. 3). Consequently, there was behavioral plasticity in signaling behavior in response to a change in social environment. Moreover individuals varied in how they responded to the change in environment: removal of ID from the original model did not significantly change the fit of the model ($\chi_{2}^{2}=2.33,P=0.31$), indicating high variation among and within males in their response to a change in social environment.

When there was another male present, focal males spent slightly less of their time signaling on the carcass than when they were alone, but there was no significant main effect of selection regime on the proportion of time spent signaling on the carcass (Table 1, Fig. 3A, B), and the interaction between G (selection regime) and E (social environment) was nonsignificant, as was the interaction between social environment and focal male body size (Table 1). However, there was a significant, positive effect of the body size of focal males on the proportion of time spent signaling on the carcass, with larger males spending more time signaling on the carcass than smaller males (Table 1).

Focal males also spent considerably less time signaling off the carcass (“satellite” behavior) when a competitor was present compared to when alone (Fig. 3C, D), but as for on‐carcass signaling behavior, selection regime, either as a main effect or in interaction with the social environment, had no effect on time spent signaling (Table 1). In contrast to on‐carcass signaling behavior, however, time spent signaling off the carcass was not related to the body size of focal males, either as a main effect or as an interaction with social environment (Table 1).

### EFFECT OF VARIATION IN QUALITY OF SOCIAL ENVIRONMENT ON SIGNALING BEHAVIOR

**Figure 4:**
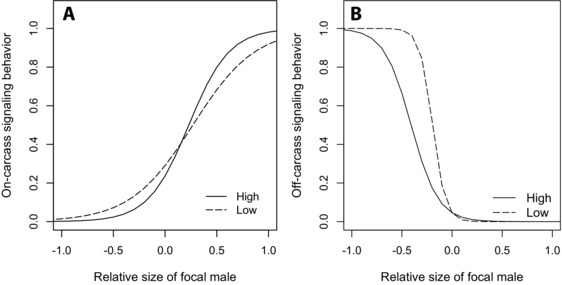
Proportion of time spent signaling on (A) and off (B) the carcass in relation to the quality of the social environment experienced by focal males (size relative to that of a competitor). Lines correspond to logistic regression models fitted to relative body size for each selection regime.

During interactions with competitors, the proportion of time spent signaling on the carcass by focal males depended upon an interaction between selection regime (G) and the size of the focal male compared to that of his rival (R) (Table 2): focal males from lines selected for high mating rates were more sensitive to variation in the quality of the social environment they experienced, being more likely to spend time signaling on the carcass when larger than their competitor, but less likely to signal on the carcass when smaller than males from lines selected for low mating rate (Fig. 4). However, there was no overall difference in the proportion of time spent signaling on the carcass between males from different selections lines (main effect of selection regime; Table 2). The quality of social environment experienced by males (i.e., the size of the focal male relative to that of his rival) was the primary determinant of on‐carcass signaling behavior: the larger the focal male compared to his rival, the greater the proportion of time spent signaling on the carcass (Table 2, Fig. 4A).

**Table 2:** Mixed model analyses effect of variation in quality of social environment on signaling behavior

On‐carcass signaling behavior		
	$x_{1}^{2}$	*P*‐value
Relative body size (R)	459.1	<0.0001
Selection regime (G)	3.354	0.067
R × G	53.27	0.0004
Off‐carcass signaling behavior		
Relative body size (R)	492.3	<0.0001
Selection regime (G)	0.001	0.972
R × G	76.23	<0.0001

*x* ^2^ Values to test the significance of the fixed effects were obtained by log‐likelihood ratio test following sequential stepwise removal of terms.

A significant interaction between selection regime and relative competitive ability was also found for off‐carcass signaling behavior (Table 2). However, in contrast to on‐carcass signaling behavior focal males from lines selected for low mating rates were more sensitive to variation in their relative competitive ability than males from high lines (Fig. 4B). There was no overall difference between selection lines in the amount of time spent signaling off the carcass, but relatively small focal males were much more likely to engage in signaling off the carcass than larger focal males (Table 2).

## Discussion

Our experiment showed that both the proportion of time that males spent on the carcass and the proportion of time spent on signaling behaviors (on‐ and off‐carcass) was plastic in response to the introduction of a rival competitor. As predicted this behavioral plasticity in response to a change in social environment was mediated by body size, with larger males investing more time in resource‐holding behaviors (both in terms of overall activity time and signaling on the carcass) than small males. There was no G × E for plasticity of signaling behavior (either on‐ or off‐carcass) in response to the introduction of a rival male, although in both cases the effects were only marginally nonsignificant. However, there was a G × E for plasticity of time spent active on the carcass (resource‐holding behavior), with males from lines selected for high repeated mating rates increasing and males from low lines decreasing overall activity on the carcass in response to the introduction of a competitor. Moreover, there was a G × E underlying the responses of males to variation in the quality of the social environment that they experienced: males from lines selected for high mating rates did not differ from low line males in either the proportion of time spent signaling on‐ or off‐carcass, but they were more sensitive to variation in the quality of the social environment when signaling on the carcass and less sensitive when signaling off the carcass. Thus, the reproductive tactics of *N. vespilloides* burying beetles are behaviorally plastic, primarily mediated by individual differences in body size among males, and there is genetic variation underlying this behavioral plasticity.

Population‐level plasticity depends upon individual‐level plasticity, but the latter cannot be inferred from determining plasticity at the level of the population (Nussey et al. 2007). This is apparent in our study, as all behaviors quantified showed individual differences in plasticity, with plasticity at the population level absent for general activity on the carcass, but present for signaling both on and off the carcass (Figs. 1, 2, 3). The absence of population‐level plasticity in on‐carcass activity occurs because small males reduced and large males increased the amount of time on the carcass when a competitor was introduced. As a result there was no overall change in behavior at the population level (Fig. 1). In contrast, there was an overall population‐level change in on‐carcass signaling, with males reducing the amount of time signaling overall when a competitor was introduced. Nevertheless, larger males signaled more than smaller males overall in both the absence and presence of competitor, with a marginally nonsignificant tendency for larger males to change their behavior less with the addition of a competitor than small males did. In contrast, although there was substantial individual‐level and population‐level plasticity in off‐carcass signaling behaviors, these were not mediated by body‐size effects. Focal males of all sizes responded to the presence of a competitor by reducing to a very low level the amount of time spent signaling off the carcass (Fig. 3C, D). This change appeared to be largely a consequence of an overall reduction in time spent signaling (as opposed to other behaviors) in a social context, especially off‐carcass, which is associated with a satellite strategy (Eggert and Müller 1989b; Eggert 1992). Focal males spent more time on the carcass than nonfocal competitor males, presumably because the focal males were the resource holders as they arrived first at the carcass (Hopwood et al. 2014; Lee et al. 2014). As a result the effect of focal male size on signaling behavior may have been partially obscured by this “residency” effect. Nonetheless, our results show that the plasticity of male signaling and overall activity in response to a change in the social environment was complex, as found in other behavioral plasticity studies (e.g., Westneat et al. 2011; Bretman et al. 2014), with the size of the focal male involved playing an important role in determining patterns of plasticity.

Our results confirm that relative competitive ability in burying beetles is largely determined by body size (Otronen 1988; Hopwood et al. 2013, 2014; Lee et al. 2014), which is itself primarily determined by parental response to variation in the resources available to beetles during the larval stage (i.e., carcass size; Bartlett and Ashworth 1988; Eggert and Müller 1997). Therefore, behavioral plasticity of signaling behavior in male *N. vespilloides* also depends upon developmental plasticity of individuals in response to variation in carcass size. This combination of current and past environments on the plasticity of behavior is thought to be common, but is rarely shown (Dingemanse and Wolf 2013).

Along with variation in the size of the resource required for reproduction, the availability of carcasses in nature is stochastic, and varies temporally and spatially (Eggert and Müller 1997). Such temporal and spatial variation, in combination with competition for resources, has been posited as an adaptive mechanism underlying individual differences in plasticity independent of state‐dependent effects (Dingemanse and Wolf 2013). The benefits of plasticity are thus negatively frequency dependent due to costs of plasticity, which favors the coexistence of both plastic and nonplastic individuals (Dingemanse and Wolf 2013). Beetles that are more plastic in their behavior may do best when there is high uncertainty about the social environment that individuals are likely to experience in competition for carcasses, for example, but lose out to individuals that are less plastic when social environmental conditions are more predictable (e.g., when carcass availability is either very low or very high). It is not known what the costs of plasticity are (if any) for burying beetles, but more generally the costs of maintaining a plastic response are expected to lead to the evolution of reaction norms that increase the fit of individuals to the more frequently occurring environments (Pigliucci 2005; Nussey et al. 2007). Our results indicate that the social environment that individuals experience depends largely on the (relative) size of the focal beetles involved (see also Hopwood et al. 2014).

The responsiveness of males to variation in the social environment they experienced depended on genetic background, indicating that behavioral plasticity evolves as a correlated response to selection on mating behavior: We found that males showed context‐dependent social sensitivity in their signaling behavior in relation to their selection regime. This suggests that selection for increased investment in repeated mating rate, a paternity assurance trait (Alcock 1994; House et al. 2008), leads to increased social sensitivity in paternity assurance contexts. Under competition from rivals dominant, resource‐holding males increase their repeated mating rate (Sakaluk and Müller 2008). The greater responsiveness we see for on‐carcass signaling behavior of males from lines selected for high mating rate (i.e., selection for greater investment in mating traits) to variation in the quality of the social environment they experience is similar to that found by Sih et al. (2014) for more active aggressive water striders (which mate at higher frequencies than less active aggressive males) and is likely to be adaptive, as the social environment experienced by burying beetles in competition for carcasses is dynamic and unpredictable (Hopwood et al. 2014). If selection favors increased investment in behaviors that protect paternity, such as repeated mating, it implies that competition for mates is likely to be strong. Given the importance of body size in determining success in direct contests over breeding resources in *N. vespilloides*, and the costs involved in engaging in contests that are likely to be lost (Otronen 1988), this may lead to fine‐tuned social sensitivity in the behavioral expression of reproductive tactics in relation to social context (i.e., when protecting paternity): males need to accurately assess their social situation to maximize their reproductive success (Bretman et al. 2011a). Such plasticity in mating behavior in response to social context has also been demonstrated in other species. For example, in the fruit‐fly *Drosophila melanogaster* mating duration of males depends on the level of competition experienced (Bretman et al. 2009, 2012), and males use a variety of different cues to detect rivals and modify responses, and thereby enhance fitness (Bretman et al. 2011b). Our results are in line with these results in *Drosophila*, but rather than using multiple cues burying beetles appear to primarily rely on a single cue, relative size difference, to influence reproductive tactics.

## Conclusion

We provide novel evidence that alternative behavioral reproductive tactics are sensitive to social context in *N. vespilloides*, and that this sensitivity is dependent upon variation in the genetic background of individuals. In doing so our experiments reveal social environment induced variation in the switch points and reaction norms of the expression of behavioral reproductive tactics in burying beetles: males selected for high investment in mating traits that protect paternity were more plastic in their behavioral response to variation in the quality of the social environment that they experienced when they were dominant than when subordinate. In contrast, males selected for low investment in mating traits were more plastic in behavior when subordinate. This shows that the plasticity and social sensitivity of behavioral reproductive tactics can evolve in response to selection on investment in mating traits: males that have more sex are more insecure.

## Supporting information

Supplementary Material
