# Supplementary material for: Behavioral plasticity and G × E of reproductive tactics in Nicrophorus vespilloides burying beetles
Source: Evolution. 2015 Mar 10;69(4):969–78. doi: 10.1111/evo.12619 (PMC5024017; doi:10.1111/evo.12619)
Supplement: Supplementary file 1 — Supplementary Material [file EVO-69-969-s001.pdf]

Artificial selection experiment

Methods

To confirm the effectiveness of our selection regime on mating rate after 12 generations (see Head et al. 2014 for data up to generation 7), we estimated realized heritabilities by least square regression (OLS) of the cumulative response (R_c) on the cumulative selection differential (S_c) forced though the origin (Falconer and Mackay 1996) within each replicate selection line. Our artificial selection experiment included a set of control populations, allowing removed common environmental effects from the total response of selection (Hill 1972). We measured the response to selection each generation using both the divergence between high and low selection lines regardless of control lines. As our selection experiment was replicated, we estimated the sampling variance of the average realized h^2 from the variance among replicated lines (Falconer and Mackay 1996).

Results

Mating rate showed a response to both upward and downward selection, as illustrated by the increasing divergence of high and low selection lines across generations (Fig. S1). Realized heritabilities were similar in magnitude for both high and low line males (Table S1). After 12 generations of divergent selection on mating rate, burying beetles from high lines mated almost twice as often as burying beetles from low lines ($F_{1,235}$ = 37.17, p = 0.025; Fig. S1). There was no effect of the selection regime on male body size (High 1: 0.52mm ± 0.06 (CI), High 2: 0.51mm ± 0.06 (CI), Low 1: 0.50mm ± 0.06 (CI), Low 2: 0.51mm ± 0.07 (CI); $F_{1,300}$ = 0.552, p = 0.593).

Table S1: Realized heritability in replicate lines, after 12th generations of divergent artificial selection on mating rate. The regression shows the Cumulative Response of Selection (R_c) against the Cumulative Selection Differential (S_c), forced through the origin.

	Ordinary Least-Square (OLS) regression		
Replicate	Upward ± SE	Downward ± SE	Divergence ± SE
1	.07 ± .003	.01 ± .003	.06 ± .002
2	.08 ± .003	.12 ± .003	.06 ± .002
Pooled	.07 ± .002	.11 ± .002	.06 ± .002
Mean	.07 ± .003	.11 ± .003	.06 ± .002

Figure S1: Direct response of mating frequency to upward and downward selection over 12 generations. Mating rate is plotted against the absolute cumulative selection differential, dots represent the mean value at each generation and lines show fitted regressions. The slopes of these regressions correspond to the realized heritabilities (absolute values) shown in table S1.

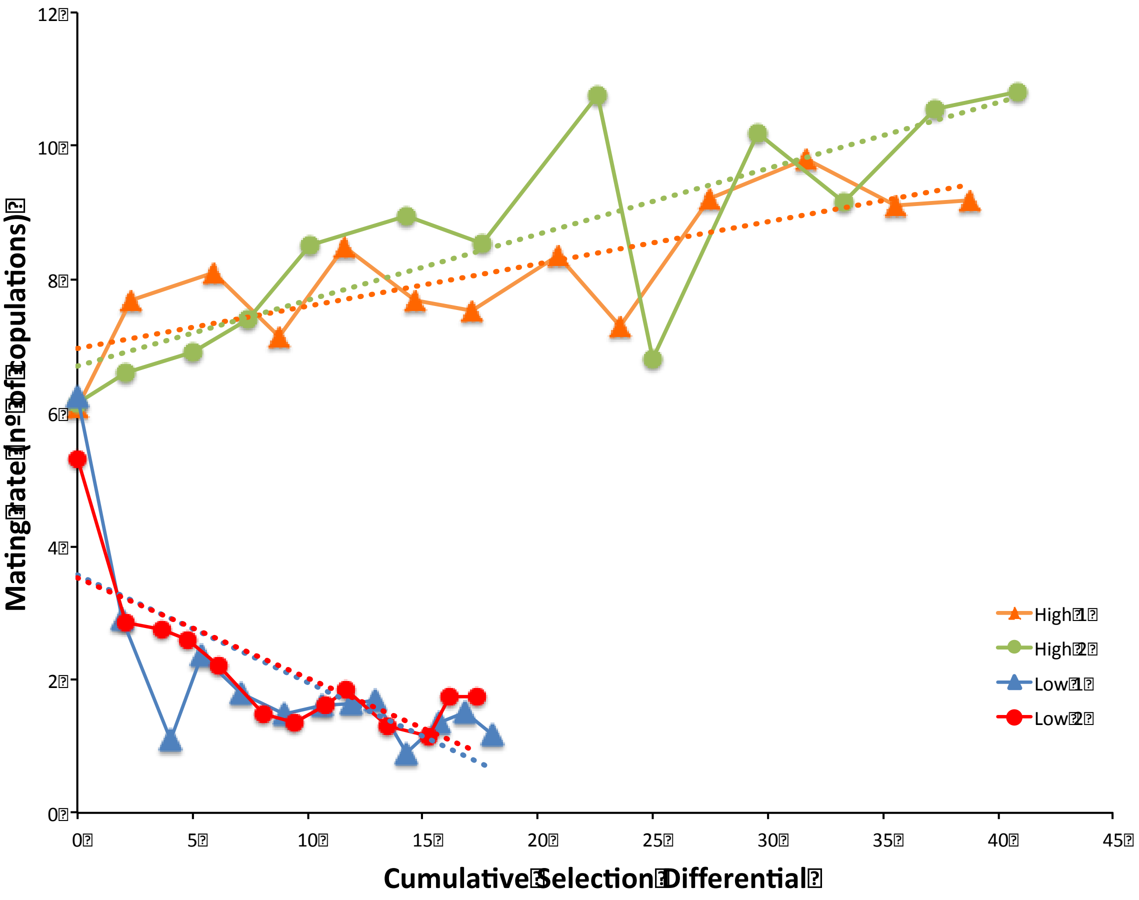

References

Falconer, D. S., and T. F. C. Mackay. 1996. Introduction to quantitative genetics. 4th ed. Pearson, London.

Head, M. L., C. A. Hinde, A. J. Moore, and N. J. Royle. 2014. Correlated evolution in parental care in females but not males in response to selection on paternity assurance behaviour. Ecology Letters 17:803-810.

Hill, W. G. 1972. Estimation of realised heritabilities from selection experiments . I . Divergent selection. Biometrics 28:747–765.
